# Aspirin Affects MDA-MB-231 Vesicle Production and Their Capacity to Induce Fibroblasts towards a Pro-Invasive State

**DOI:** 10.3390/ijms241512020

**Published:** 2023-07-27

**Authors:** Rafaela de Assiz Louback, Karina Martins-Cardoso, Luzineide W. Tinoco, Federica Collino, Ana Paula D. N. de Barros, Anneliese Fortuna-Costa, Robson Q. Monteiro, Maria Isabel Doria Rossi, Rafael Soares Lindoso

**Affiliations:** 1 Institute of Biomedical Sciences, Federal University of Rio de Janeiro, Rio de Janeiro 21941590, Brazil; rafaelalouback@gmail.com (R.d.A.L.); apdantas@gmail.com (A.P.D.N.d.B.); anneliese.fortuna@gmail.com (A.F.-C.); 2 Clementino Fraga Filho University Hospiyal, Federal University of Rio de Janeiro, Rio de Janeiro 21941913, Brazil; 3 Institute of Medical Biochemistry Leopoldo de Meis, Federal University of Rio de Janeiro, Rio de Janeiro 21941590, Brazil; kmcardosos@bioqmed.ufrj.br (K.M.-C.); robsonqm@bioqmed.ufrj.br (R.Q.M.); 4 Laboratory for Analysis and Development of Enzyme Inhibitors, Natural Products Research Institute, Federal University of Rio de Janeiro, Rio de Janeiro 21941590, Brazil; luzitinoco@gmail.com; 5 Department of Clinical Sciences and Community Health, University of Milano, 20122 Milan, Italy; federica.collino@unimi.it; 6 Laboratory of Translational Research in Paediatric Nephro-Urology, Fondazione IRCCS Ca’ Granda-Ospedale Maggiore Policlinico, 20122 Milan, Italy; 7 Pediatric Nephrology, Dialysis and Transplant Unit, Fondazione IRCCS Ca’ Granda-Ospedale Maggiore Policlinico, 20122 Milan, Italy; 8 Carlos Chagas Institute of Biophysics, Federal University of Rio de Janeiro, Rio de Janeiro 21941902, Brazil; 9 National Center of Science and Technology for Regenerative Medicine/REGENERA, Federal University of Rio de Janeiro, Rio de Janeiro 21941902, Brazil

**Keywords:** aspirin, extracellular vesicles, cancer-associated fibroblasts, breast cancer, spheroid

## Abstract

Long-term administration of aspirin (ASA, acetylsalicylic acid) in oncogenic patients has been related to a reduction in cancer risk incidence, but its precise mechanism of action is unclear. The activation of cancer-associated fibroblasts (CAFs) is a key element in tumor progression and can be triggered by cancer-derived extracellular vesicles (EVs). Targeting the communication between cancer cells and the surrounding tumor microenvironment (TME) may control cancer progression. Our aim was to investigate the effect of ASA on breast cancer cells, focusing on EV secretion and their effect on the biological properties of CAFs. As a result, ASA was shown to reduce the amount and alter the size distribution of EVs produced by MDA-MB-231 tumor cells. Fibroblasts stimulated with EVs derived from MDA-MB-231 treated with ASA (EV-ASA) showed a lower expression of alpha-smooth muscle actin (α-SMA), matrix metalloproteinase-2 (MMP2) but not fibroblast activation protein (FAP) in respect to the ones stimulated with EVs from untreated breast cancer cells (EV-CTR). Furthermore, invasion assays using a three-dimensional (3D) fibroblast spheroid model showed reduced MDA-MB-231 invasion towards fibroblast spheroids pretreated with EV-ASA as compared to spheroids prepared with EV-CTR-stimulated fibroblasts. This suggests that ASA partially inhibits the ability of tumor EVs to stimulate CAFs to promote cancer invasion. In conclusion, ASA can interfere with tumor communication by reducing EV secretion by breast tumor cells as well as by interfering with their capacity to stimulate fibroblasts to become CAFs.

## 1. Introduction

Aspirin (ASA, acetylsalicylic acid) is a widely used drug due to its low toxicity, low cost, and easy access. It has anti-inflammatory, analgesic, antipyretic, and anti-platelet actions [1]. Meta-analysis observational studies that have been published recently underline the beneficial effect of ASA intake for many types of cancer, resulting in a reduction of tumor progression and metastasis and consequently increasing patient survival [2,3,4,5,6,7,8,9,10,11,12,13,14]. Specifically, in breast cancer patients, the first clinical evidence for this effect was published in a study that found that women who regularly took aspirin at least twice a week in the years following cancer diagnosis showed a reduction in the relative risk of breast cancer recurrence and mortality [9]. In vitro studies also confirmed that ASA may exhibit antitumor activity in breast cancer cells by impairing important pathophysiological events and inducing cell death [15,16,17]. Besides its direct action in the inhibition of cyclooxygenases, it has been described that aspirin can have many other effects. Among them, ASA can modulate TGF-ß1 secretion, leading to apoptosis of tumor cells and immunomodulatory effects via the direct regulation of the transcription factor nuclear factor-kappa B (NF-kappa B) activation [18,19,20]. ASA can also inhibit the pro-tumoral IncRNA-miRNA-mRNA signaling pathways, inducing apoptosis and DNA repair system regulation [3,18,21,22,23], beyond the acetylation of other proteins [24,25,26]. Despite the evidence of ASA antitumor activity, the precise molecular mechanism that acts to benefit cancer patients remains to be further investigated.

It is well-established that tumor biology is regulated not only by the genetic and epigenetic events of tumor cells but also through the participation of a complex system of cooperation between the tumor and other cell types that constitutes what is called the tumor microenvironment (TME) [27,28,29,30]. The main dominant non-tumor cells in TM are cancer-associated fibroblasts (CAF), which have been considered a tumor-promoting component of the microenvironment as they regulate many of the conditions required for tumor development, such as support for tumor cell proliferation, invasion, and metastasis. However, CAFs are a highly heterogeneous cell population that may behave as a tumor-promoting or tumor-restraining component [31,32,33,34,35,36,37]. Within the tumor stroma, different subtypes of CAFs exist and are interconvertible depending on microenvironmental cues [37].

Among the molecular mediators responsible for the tumor influencing the surrounding non-tumor stromal cells to behave as CAFs, EVs have been described as fundamental [38,39,40]. EVs are spherical particles limited by a lipid bilayer that carry various types of molecules derived from the cell of origin and are important in many pathophysiological events, including tumor development [41,42,43,44,45]. EVs are classified according to their dimension and biogenesis mechanism into exosomes, microvesicles, and apoptotic bodies [41,46,47]. Among those, exosomes and microvesicles have been deeply studied as mediators of intercellular communication. The microvesicles (average size 200–800 nm) are directly derived from plasma membrane protrusions, while exosomes (average size 30–150 nm) are derived from endosomal machinery [48]. In cancer, EVs carry molecules such as proteins, lipids, RNAs (mRNAs, miRNAs, and lnc-RNAs), and DNA fragments that can change the TME [48].

Aspirin has been described as causing a decrease in the number of platelet-derived EVs both in healthy individuals [49,50] and in patients with coronary artery disease [51,52]. Moreover, in such patients, endothelial cells, erythrocytes, monocytes, and vascular wall-derived exosomes were drastically reduced. Recently, it was demonstrated that hypoxic-mediated exosomes, released by lung cancer cells treated with ASA, changed their capability in promoting proliferation, migration, and angiogenesis by a modification of their content [53]. Besides, low doses of ASA (0.2 mM in vitro and 600 µg/mL in vivo) suppressed pancreatic tumor repopulation by inhibiting the secretion of exosomes [54]. Few publications have deeply investigated ASA activity in other tumors. A recent example reported that ASA can target cancer stem cells and enhance the efficacy of chemotherapy by modulating the transcription regulator SMAR1, indicating that ASA might also have therapeutic applications in other tumors [55].

In this study, we evaluated the effects of aspirin on the release of EVs by breast tumor cells. EV production, dimension, and uptake by fibroblasts were evaluated. Moreover, the impact of ASA-treated tumor cell-derived EVs on the phenotype of fibroblasts and on their capability to be activated towards a pro-invasive CAF-like state was also analyzed.

## 2. Results

### 2.1. High Concentrations of ASA Induce Tumor Cell Death In Vitro

**Figure 1:**
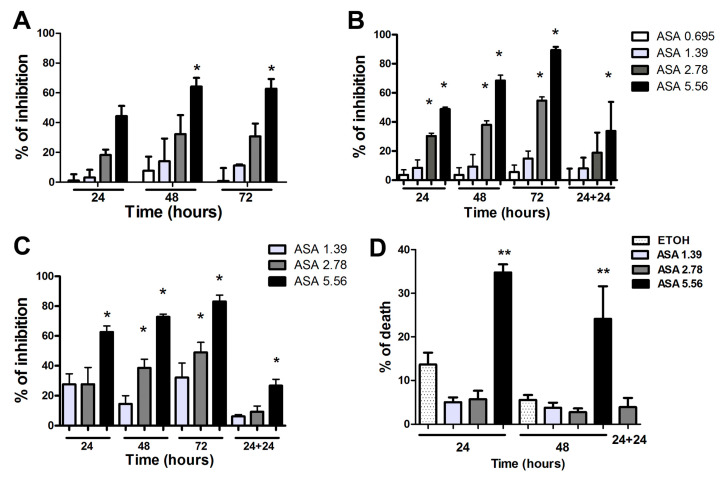
ASA reduces cell viability at high concentrations. Breast cancer tumor cell lines MCF-7 (**A**), T-47D (**B**), and MDA-MB-231 (**C**,**D**) were treated for up to 72 h with different concentrations (0.695–5.56 mM) of ASA. T-47D (**B**) MDA-MB-231 (**C**) cells were also treated for 24 h with different concentrations (0.695–5.56 mM) of ASA and then maintained for 24 h in the absence of ASA (24 + 24). (**A**–**C**) The percentage of inhibition of formazan crystal formation following reduction by MTT was calculated in relation to control cells incubated with vehicle (**D**) The percentage of MDA-MB-231 cells positive for Annexin V/PI (cell death) was evaluated by FACS analysis. Data show mean ± SEM of three independent experiments. * *p* < 0.05; ** *p* < 0.001.

ASA treatment (2.5–10 mM) was demonstrated to induce the apoptosis of a variety of human tumor cell lines, including breast cancer cell lines, in vitro [16,17]. We first evaluated the effects of different ASA concentrations (0.695, 1.39, 2.78, and 5.56 mM) and time of treatment (24, 48, and 72 h) on the viability of three human breast cancer cell lines: two estrogen receptor (ER)-positive, MCF-7 and T-47D (Figure 1A,B, respectively), and a triple-negative breast cancer cell line, MDA-MB-231 (Figure 1C,D). The higher concentration (5.56 mM) of ASA significantly reduced the viability of all breast cancer cell lines (Figure 1A–C) even after 24 h of treatment, except the MCF-7 cell line, as observed by the percentage of enzyme activity’s inhibition with respect to untreated cells. Furthermore, a significant reduction in T-47D cell viability was observed after 24 h of treatment with a concentration of 2.78 mM (Figure 1B). A similar effect was observed in MDA-MB-231 only after 48 h of incubation with the same concentration of ASA (Figure 1C). Interestingly, the removal of ASA from the culture medium after 24 h allowed the recovery of both cell lines within 24 h of culture (24 + 24 condition) under a 2.78 mM concentration but not 5.56 mM. The lowest concentrations (0.695 and 1.39 mM) did not significantly affect the viability of all the cells tested. To confirm the effect of ASA on MDA-MB-231 cells, we performed a cell death assessment by Annexin V/PI assay using the same culture setting (Figure 1D). We observed that only the highest concentration (5.56 mM) of ASA induced death in MDA-MB-231 cells after 48 h of treatment. Thus, the concentration of 2.78 mM ASA applied for 24 h and then removed (24 + 24 h) was chosen as the best nontoxic setting to be used in the following assays. No difference in viability was observed between untreated MDA-MB-231 cells and cells treated with ETOH (vehicle). Additionally, we analyzed if the proliferation rates of MDA-MB-231 were altered by ASA and its removal after 24 h by evaluating the proliferation marker Ki-67 expression by FACS analysis (Supplementary Figure S1). MDA-MB-231 cultured for 24 h and 24 + 24 h with 2.78 mM of ASA or control vehicle (ETOH) did not present any difference in the proliferation rate.

### 2.2. ASA Decreases EV Production by Breast Tumor Cells

**Figure 2:**
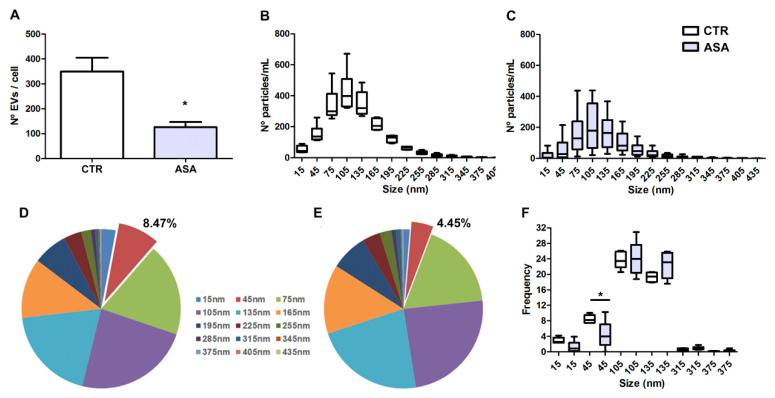
ASA promotes changes in EV production by breast cancer cells. MDA-MB-231 derived EVs were isolated from the conditioned medium (CM) of cells incubated in the presence, or not, of 2.78 mM of ASA for 24 h (24 h). The EVs were collected 24 h after ASA removal. (**A**) Quantification of EVs secreted by cells treated (EV-ASA) or not (EV-CTR) with ASA. Data represent mean ± SEM of 5 (CTR) and 13 (ASA) independent experiments. * *p* = 0.0002. (**B**,**C**) Distribution and average size profile of EV-CTR (**B**) and EV-ASA (**C**) isolated from CM. Data show a median, maximum, and minimum of six independent experiments. (**D**,**E**) Pie chart showing the size distribution of each particle fraction secreted by control (**D**) and ASA-treated (**E**) cells. (**F**) Data represent the frequency of the different range size concentrations of EVs in CTR (white) and ASA (grey) conditions. The difference in the percentage of the 45 nm fraction was significant (* *p* < 0.05).

We then investigated the effect of ASA on the release of EVs by MDA-MB-231. The MDA-MB-231 were maintained in culture with 2.78 mM ASA (EV-ASA) or not (EV-CTR) for 24 h. After the removal of ASA, tumor cells were maintained for an additional 24 h in culture, and EVs were then isolated. ASA treatment induced a significant reduction in the number of EVs released by MDA-MB-231 in comparison to the untreated condition (Figure 2A).

Regarding the size of EVs, we observed that the distribution profile was very similar between conditions, with an average size of 120 nm and 125 nm (EV-CTR and EV-ASA, respectively) (Figure 2B,C). However, by comparing the percentage of the size of the different fractions separately with respect to the total, we observed a decrease of the EV population around 45 nm after ASA treatment (Figure 2D,E). Altogether, these data indicate that a non-toxic ASA concentration is able to reduce EV production by the human breast cancer cell line, MDA-MB-231.

### 2.3. EVs from ASA-Treated and Untreated Breast Tumor Cells Promote Distinct Effects on Human Skin Fibroblasts (HSF)

To assess the changes promoted by ASA on the effects of tumor-derived EVs, we initially evaluated their uptake by HSF. Such cells were incubated with labeled tumor EVs for different periods of time, as described [56]. However, after overnight (ON) and 24 h of incubation, we observed a punctiform pattern on the fibroblast cytoplasm in cells that received EVs derived from both treated and untreated tumor cells (Supplementary Figure S2), indicating the presence of EVs accumulated into the cells after internalization. It suggests that the treatment with ASA does not interfere with the EVs’ uptake by HSF.

**Figure 3:**
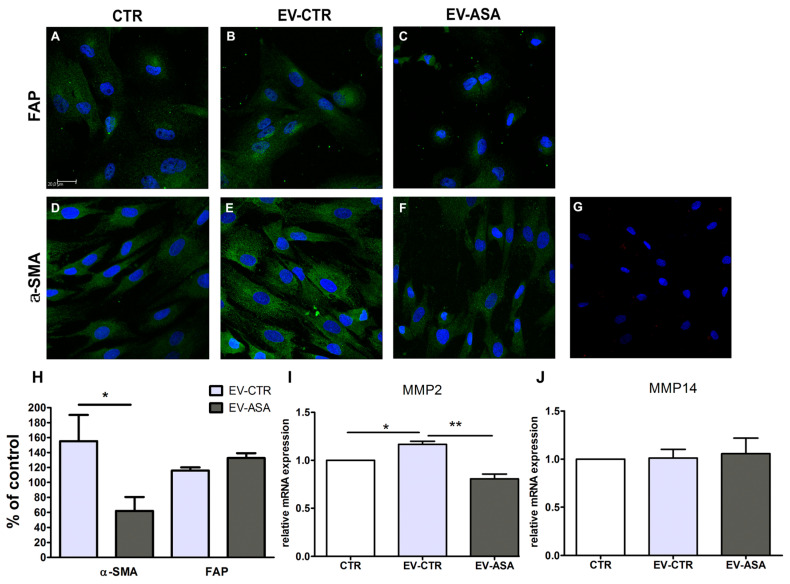
ASA modifies the profile of EV-stimulated fibroblasts. Human fibroblasts were stimulated with EVs released from MDA-MB-231 treated with 2.78 mM of ASA (EV-ASA) or not (EV-CTR) (24 + 24 h treatment). Expression of FAP (**A**–**C**, green) and α-SMA (**D**–**F**, green) in control (CTR) unstimulated cells (**A**,**D**) and EV-CTR (**B**,**E**) or EV-ASA (**C**,**F**) stimulated cells. (**G**) Secondary antibody control. Nuclei were stained with DAPI (blue). Bar = 20 µm. Representative images of three independent experiments. (**H**) Fluorescence intensity of α-SMA and FAP in regions of interest, adjusted to the percentage of control. (**I**,**J**) Gene expression of metalloproteinases-2 (MMP-2; **I**) and 14 (MMP-14; **J**) were analyzed by quantitative RT-PCR. GAPDH was used as the reference gene. Relative expression of mRNA was calculated using the ΔΔCT method. Data represent mean ± SEM of three independent experiments. * *p* < 0.05 ** *p* < 0.001.

Based on these data, we investigated the effects of EVs produced by MDA-MB-231 cells treated or not with ASA (EV-ASA and EV-CTR, respectively) on HSF. We evaluated the expression of two markers upregulated in CAF: FAP and α -SMA. Under the culture conditions used, HSF cells already showed basal expression of both markers (Figure 3A,D). This could be associated with the response of HSF to standard culture conditions that can modulate some cellular processes (e.g., cytoskeleton organization, migration, and proliferation), as previously reported by other groups [57,58,59,60]. Likewise, HSF stimulated with EV-CTR or EV-ASA was also positive for these two markers (Figure 3B–F). Analysis of fluorescence intensity in regions of interest showed a significant increase in α-SMA expression by HSF cells incubated with EV-CTR compared to unstimulated cells (CTR). However, such an increase was not observed in HSF stimulated with EV-ASA. No differences were observed in FAP expression by HSF cells in any condition (Figure 3H).

We have also evaluated the expression of the pro-invasive metalloproteinases (MMPs), MMP2 and MMP14 [61,62]. Fibroblasts treated with EV-CTR showed a mild but significant increase in MMP2 expression with respect to unstimulated cells. However, MMP2 mRNA levels were significantly lower when fibroblasts were treated with EV-ASA (Figure 3I). On the contrary, the same was not observed for MMP14 mRNA levels, which showed no differences in the EV-treated HSF cells (Figure 3J).

To understand if part of the effects observed in HSF could also be given by reminiscent free aspirin in the conditioned medium (CM) of ASA-treated MDA-MB-231, the presence of ASA was investigated by nuclear magnetic resonance (NMR). Data show that after 24 h of incubation with MDA-MB-231, no trace of ASA was observed, possibly due to its hydrolyzation (Supplementary Figure S3). This data indicates that the observed changes in HSF were not directly mediated by ASA but instead by the EVs secreted by the tumor cell.

### 2.4. EVs Derived from ASA-Treated Breast Tumor Cells Are Unable to Induce A Pro-Invasive Behavior in Fibroblasts

We then aimed to evaluate the capacity of EV-derived tumor cells to induce a pro-invasive phenotype, one of the main biological properties exerted by CAFs [56]. Based on a previous study [63], we initially quantified the tumor cell invasion using a three-dimensional (3D) spheroid model that secretes and self-assembles ECM components. To that end, both triple-negative breast cancer cell lines, MDA-MB-231 and MDA-MB-468, were used, showing the capability to invade HSF spheroids (Supplementary Figure S4A–C). The invasive capacity of MDA-MB-231 cells was significantly higher after 24 and 48 h of co-culture with respect to the MDA-MB-468 cells (Supplementary Figure S4C). On the contrary, the non-invasive MCF-7 cell line was unable to invade the periphery of HSF spheroids after 72 h of co-culture (Supplementary Figure S4D).

**Figure 4:**
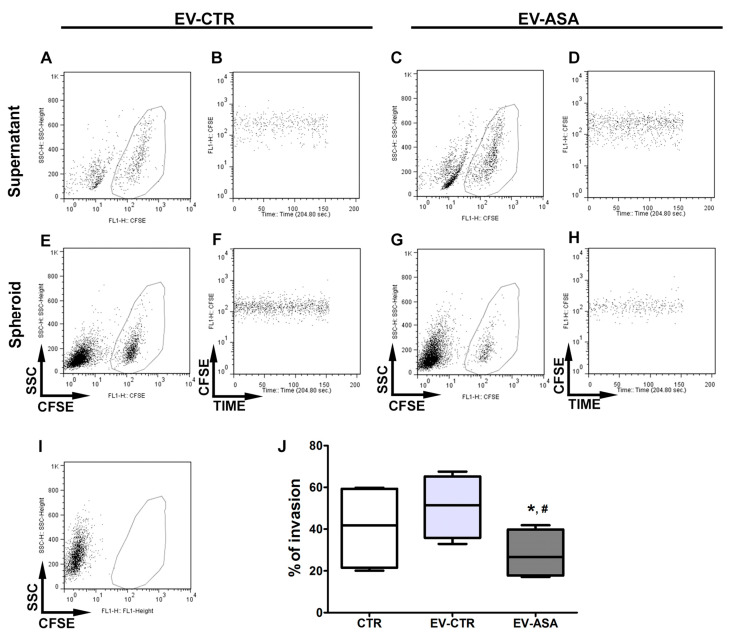
EVs derived from ASA-treated breast tumor cells block the phenotypical switch of fibroblasts into CAF and their tumor pro-invasive properties. Spheroids made of unstimulated (CTR), EV-CTR, or EV-ASA stimulated fibroblasts were cultured for 24 h with CFSE-labeled MDA-MB-231 cells. The supernatant was harvested, and the spheroids were enzymatically dissociated. The amount of CFSE + events was evaluated by flow cytometry, with time as the acquisition parameter. (**A***–***I**) Dot plots showing (**A**,**C**,**E**,**G**) selected CFSE + events and (**B**,**D**,**F**,**H**) its distribution over the acquisition time in (**A***–***D**) supernatants and (**E***–***H**) spheroids. (**I**) Dot plot of spheroids maintained in the absence of labeled tumor cells, showing no CFSE + events. (**J**) Quantification of invasion, calculated as the percentage of CFSE + events inside the spheroids in relation to the total of CFSE + events (supernatant + spheroid). Data show mean ± SEM of the average of five independent experiments conducted in duplicate or triplicate. *, # *p* < 0.05 vs. EV-CTR and CTR, respectively.

The capacity of MDA-MB-231 cells to invade spheroids was then tested in unstimulated HSF and HSF cells stimulated with EV-ASA or EV-CTR. After 24 h of co-culture, MDA-MB-231 cells invaded significantly less the spheroids formed by HSF previously stimulated with EV-ASA compared to those that were stimulated with EV-CTR (Figure 4). Interestingly, a slight increase, which did not reach statistical significance, was observed comparing the invasion of spheroids formed by EV-CTR-stimulated fibroblasts with that of unstimulated ones (Figure 4).

## 3. Discussion

In the past few years, there have been a growing number of studies linked aspirin intake with benefits for cancer patients. Still, the precise mechanism involved in the antitumor effects of aspirin is not clear. In this study, we described that aspirin could not only reduce the number of EVs produced by breast tumor cells but also alter the breast cancer’s ability to stimulate fibroblasts’ acquisition of the CAF phenotype.

The sensitivity of ASA has been correlated to the level of malignancy in tumor cells [16,17]. Indeed, in our data, we show that the less aggressive T47-D breast tumor line was more sensitive to ASA treatment than the metastatic MDA-MB tumor cells. This was in line with a few in vitro [16] and in vivo [64] studies supporting a more beneficial effect of ASA treatment for ER+ breast cancer patients. However, no published data from prospective clinical studies are present.

In our hands, aside from the toxicity effect that ASA can cause on tumor cells, aspirin presented a significant capability to reduce the secretion of pro-invasive EVs by tumor cells. The EVs have been considered a key element in tumor growth, invasion, and immune escape [65,66]. As an example, Ozawa et al. showed that EVs derived from HCC1806 squamous carcinoma cells supported the proliferation and drug resistance ability of non-tumorigenic MCF10A breast cells, followed by enrichment of cancer-related miRNAs in the EVs [67]. Additionally, exosomes derived from triple-negative breast cancer (TNBC) were shown to increase collagen contraction, migration, and CAF molecular markers in normal fibroblasts. Such effects were caused by the synergetic action of miRNAs (miR-185-5p, miR-652-5p, and miR-1246) present in the exosomes [68]. It is worth mentioning that the decrease in EV production occurs after ASA withdrawal and that there were no remaining traces of aspirin in the supernatant, supporting the specificity of the EVs’ effect. Such effect is consistent with the inhibitory effects on cancer in patients who use ASA on alternate days [9].

The precise mechanism involved in such inhibition is not clear; however, ASA is known for its protein acetylation properties, which could interfere with the regulation of EV biogenesis [26]. One example is the role of ASA in the regulation of microtubule rearrangement [69], which is known to participate in the transport of multivesicular bodies (MVBs) to the plasma membrane, resulting in exosome secretion [47]. Besides, the analysis of the concentration of EVs of different sizes showed that ASA treatment changed the dimension profile of the secreted EVs by tumor cells. In our model, only small vesicles (around 45 nm) were less enriched in the EVs released by tumor cells submitted to ASA treatment. A similar effect of ASA on changing a specific subpopulation of EVs was reported in lung tumor cells, precisely in exosomes induced by hypoxia [53].

Our experimental model, using the same concentration of EV-CTR and EV-ASA, shows that ASA not only modulates EV secretion but also their biological properties. The reduction in -SMA protein levels by EV-ASA emphasized the capability of ASA to abrogate the pro-invasive properties observed in fibroblasts incubated with EV-CTR. On the contrary, the expression of FAP, a molecule involved in epithelial-to-mesenchymal transition and tumor invasion [70], was unvarying after both EV-CTR and EV-ASA treatment, showing the activation or reduction of a selective pathway mediated by tumor-derived EV. Additionally, as CAFs secrete a wide variety of MMPs [33,34], we evaluated their expression in fibroblasts after EV treatment. Incubation with tumor-derived EVs induced a significant upregulation of MMP2 gene expression. MMP2 transcript was reduced in fibroblasts treated with ASA-EV, while no modification in MMP9 expression was observed with both CTR-EV and ASA-EV. The same increase in MMP2 has been observed in mesenchymal stromal cells stimulated by tumor-derived EV [56]. Besides, it was described that fibroblasts stimulated with ovarian tumor-EV had a higher secretion of the inactive form of MMP2 [71]. Interestingly, the existence of myofibroblast and non-myofibroblast CAFs was recently observed in the TME. Non-myofibroblast CAFs show low expression of α-SMA and an inflammatory profile, while myofibroblast CAFs show high levels of α-SMA, which correlates with increased secretion of MMPs [35].

Furthermore, because of the reduced capacity of EV-ASA to support fibroblast activation, tumor invasion was shown to be reduced in HSF spheroids previously stimulated with EV-ASA with respect to EV-CTR. Such reduction could be due to the direct interaction with the fibroblasts but also to the modification in ECM composition. For instance, CAFs modify the ECM mesh at the invasive front, leading to the migration of cancer cells [72]. Since the expression of MMP2 was shown to be increased by treatment with MDA-derived EVs, this would facilitate the digestion of ECM molecules such as collagen I, facilitating the invasion of breast tumor cells. On the other side, fibroblasts stimulated with EV-ASA did not show such an increase in MMP2 levels, restricting the invasion capacity of tumor cells.

These results clearly support the ability of ASA to modify tumor EV cargo and, afterward, block the fibroblast switch to a CAF phenotype, thus compromising their pro-invasive properties. In effect, the maintenance of normal fibroblasts exerts an inhibitory force on tumorigenesis and tumor progression [28].

In summary, our data indicate the potential of ASA to exert antitumor activity by alternating EV-mediated communication in the tumor microenvironment. Such changes are not restricted to the reduction in EV secretion but also to abrogating EV properties to stimulate CAF activation and consequently tumor cell invasion. Although further studies are necessary to understand the mechanism involved in such modulation, our data present new perspectives on ASA effects and its use in the clinic.

## 4. Materials and Methods

### 4.1. Reagents and Antibodies

Aspirin (ASA, Acetylsalicylic acid) was purchased from Sigma Aldrich Co., Saint Louis, MO, USA. A stock solution of 50 mg/mL (0.278 M) in absolute ethanol was prepared and solutions of 125, 250, 500, and 1000 µg/mL (0.695, 1.39, 2.78, and 5.56 mM, respectively) were freshly prepared in culture medium for cell viability assays [16]. Also, a solution of 500 µg/mL of ASA was freshly prepared in the culture medium for the isolation of extracellular vesicles (EVs). Unconjugated rabbit polyclonal anti-FAP (Fibroblast activation protein) and anti-αSMA (Smooth muscle α-actin) were from Abcam (Cambridge, UK). Unconjugated mouse monoclonal anti-αSMA (clone 1A4) was purchased from Sigma-Aldrich. Secondary antibodies goat anti-rabbit IgG (H + L) cross-adsorbed Alexa Fluor 488 and goat anti-mouse IgG (H + L) highly cross-adsorbed Alexa Fluor 546, and DAPI (4′,6-diamidino-2-fenilindole) were all from Thermo Fisher Scientific (Waltham, MA, USA).

### 4.2. Cell Culture Conditions

Human skin fibroblast cell line (HSF), human breast cancer cell lines MCF-7, T-47D, MDA-MB-231 and MDA-MB-468 were obtained from the Rio de Janeiro Cell Bank (BCRJ, Rio de Janeiro, Brazil). Cells were routinely cultured in Dulbecco’s modified Eagle’s medium (DMEM) supplemented with 10% fetal bovine serum (FBS, Gibco, Thermo Fisher, Grand Island, NY, USA) and antibiotics, using a combination of 100 U/mL of Penicillin G sodium and 100 μg/mL of Streptomycin, or Ciprofloxacin alone at the final concentration of 10 μg/mL, all from Sigma-Aldrich. For MTT and apoptosis assays, cells were cultured in Iscove’s modified Dulbecco’s medium (IMDM) with 0.5% FBS. For EV isolation, MDA-MB-231 cells were maintained in IMDM without FBS. For stimulation of HSF with MDA-MB-231-derived EVs, cells were cultured in DMEM supplemented with 10% EV-depleted FBS. For spheroid formation, HSF was cultured in IMDM supplemented with 20% EV-depleted FBS and antibiotics.

### 4.3. Cell Tracing

Human breast cancer cell lines MDA-MB-231, MDA-MB-468 and MCF-7 were stained with the fluorescent cell tracker CFSE (Carboxyfluorescein succinimidyl ester, Thermo Fisher) as previously described [56,63]. More specifically, cells were enzymatically dissociated, washed twice with PBS, washed once with PBS plus 0.1% bovine serum albumin (BSA, Sigma-Aldrich) and quantified. After that, cells were resuspended at the concentration of 2–5 × 10^6^ cells/mL of PBS plus 0.1% BSA and 10 μM CFSE, and incubated for 10 min at 37 °C. Then, cells were washed with PBS and 10% FBS and resuspended in IMDM with 20% EV-depleted FBS and used for the invasion assay.

### 4.4. Obtaining Fetal Bovine Serum Depleted of Extracellular Vesicles

FBS was centrifuged at 1600× *g* for 20 min. The supernatant was harvested and ultracentrifuged at 110,000× *g* (Rotor 70Ti, Beckman Coulter Optima L-90K ultracentrifuge, Brea, CA, USA) for 16 h at 4 °C. The supernatant was harvested and maintained at −20 °C.

### 4.5. MTT Assay

Cells were plated in sextuplicate in flat 96 well plates at a density of 2 × 10^4^ cells/well in IMDM supplemented with 0.5% FBS and allowed to attach overnight at 37 °C with 5% CO_2_. Cells were treated with different concentrations of ASA for up to 72 h. Control of the diluent (ETOH) and cells with no treatment were prepared. The culture medium was changed daily. At intervals of 24, 48, and 72 h, 10 µL of a stock solution (5 mg/mL in phosphate-buffered saline) of the MTT dye (3-(4,5-dimethyl-2-thiazyl)-2,5-diphenyl-2H-tetrazolium bromide; Sigma-Aldrich) was added. The plates were incubated for 3 h at room temperature and 200 µL of the solubilization solution (DMSO, dimethyl sulfoxide from Farmoterápica, Porto Alegre, RS, Brazil) were added. The absorbance was measured on a microplate reader (VersaMax™ Microplate Reader; Molecular Devices, San José, CA, USA) at a wavelength of 540 nm. The entire experiment was repeated three times.

### 4.6. Apoptosis and Proliferation Assay

MDA-MB-231 cells were plated in 25 cm^2^ flasks and after they reach 80% confluence, they were treated the same way for the MTT assay. At the end of each period of incubation, cells were enzymatically dissociated and quantified. Then, the assay was performed using an Annexin V-PI kit (BD Biosciences, San Jose, CA, USA), following the manufacturer’s instructions. Briefly, 10^5^ cells in 100 μL of Annexin Buffer Solution were incubated with 5 μL of Annexin V-FITC and Propidium Iodide (PI) for 15 min at room temperature. To evaluate the proliferative fraction, intracellular staining with the antibody anti-Ki67 was performed as previously described [73]. Briefly, MDA-MB-231 cells were cultured in the presence of 2.78 mM of ASA or ETOH, vehicle) for 24 h. The culture medium was then changed, and cells were maintained for 24 h without ASA or ETOH (24 + 24 h condition). The cells were harvested and fixed with 1% paraformaldehyde in phosphate-buffered saline (PBS), permeabilized with 70% ethanol at −20 °C, for more than 30 min and washed twice with cold staining buffer (PBS supplemented with 3% FBS and 0.1% sodium azide, Sigma-Aldrich). The cells were then incubated for 1 h on ice with the FITC-conjugated monoclonal mouse anti-Ki67 antibody (mib-1; clone B56 from BD-Biosciences). Cells were acquired by flow cytometer FACS Canto II (BD Biosciences) and analyzed by the software Diva v.9.0 (BD Biosciences or FlowJo v.9).

### 4.7. Isolation of Tumor Derived-EVs and Stimulation of HSF

MDA-MB-231 cells were cultured in 175 cm^2^ flasks until they reach 80% confluence. After that, cells were incubated with 50 mL of IMDM, without FBS, with or without ASA at a concentration of 2.78 mM. Cells were cultured for 24 h (24 h) and then, conditioned medium was harvested, and monolayer was washed with phosphate-buffered saline to remove all ASA residues. After that, were added 30 mL of IMDM without FBS or ASA plus 0.1% BSA and cells were cultured for more 24 h (24 + 24 h). Then, the conditioned medium was harvested, and cells were enzymatically dissociated and quantified in Neubauer’s chamber with Trypan Blue.

The conditioned medium harvested was centrifuged at 1600× *g* for 20 min to remove debris and subsequently centrifuged at 100,000× *g* for 2 h at 4 °C (Beckman Coulter Optima L-90K ultracentrifuge) for EV isolation. EVs were resuspended in Roswell Park Memorial Institute-1640 (RPMI, Thermo Fisher) with 1% DMSO and frozen at −80 °C. The amount and size of EVs were determined by ZetaView^®^ (ParticleMetrix, Munich, Germany). The post-acquisition parameters used were maximum size = 200 nm; minimum size = 5 nm; minimum bright = 20. The quantification data were expressed in the number of particles per cell or total amount per volume (mL), according to performed analysis.

EVs from the 24 + 24 h condition were used for HSF stimulation, following the protocol of long stimulation [56]. More specifically, cells were cultured in IMDM supplemented with 10% EV-depleted FBS and incubated with EVs from aspirin-treated or not tumor cells (EV-ASA and EV-CTR, respectively), under the proportion of 5 × 10^4^ vesicles per HSF. This stimulation was performed three times with an interval of five days between them, reaching a total of two weeks. At the end of the last stimulation, cells were enzymatically dissociated, quantified, and targeted for immunofluorescence, molecular biology and invasion assays.

### 4.8. Evaluation of Tumor-EV Incorporation by HSF

This experiment was performed based on the assay previously described [56]. First, to obtaining of labeled tumor-EV, MDA-MB-231 cells were incubated with 5 μL/mL of cell tracker VybrantDiI (Thermo Fisher) at a concentration of 1 × 10^6^ cells/mL in DMEM without FBS for 20 min at 37 °C. After that, this suspension was centrifuged at 487× *g* for 5 min. Cells were resuspended, washed twice and cultured in heated DMEM with 10% FBS. After 24 h, cells were submitted to previously described conditions of ASA treatment for EV isolation at the time of 24 + 24 h. Then, 3 × 10^4^ HSF cells were distributed on coverslips in a 24-well plate with IMDM supplemented with 10% EV-depleted FBS. After cell adhesion, the labeled tumor-EVs were added at a concentration of 5 × 10^4^ vesicles per HSF, and cells were cultured up to 24 h. After that, cells were washed and fixed with 4% paraformaldehyde for 15 min in the dark. Then, cells were washed twice with PBS, permeabilized, and incubated for 15 min with Alexa-647 conjugated phalloidin (Thermo Fisher) and DAPI. Then, the coverslips were washed twice and mounted on a slide with N-propyl-gallate. For each condition, images were taken from five random fields at 200× magnification using a Leica TCS-SPE Confocal Microscope (Leica Microsystems, Wetzlar, Germany).

### 4.9. Development of Spheroid Cell Culture and Invasion Assay

Spheroids were developed as previously reported [63]. Briefly, 200 μL of 1% agarose (Invitrogen, Waltham, MA, USA) was added per well of 96 well-round bottom plates and immediately removed. The plates were allowed to dry at RT, and 25 × 10^3^ HSF, control and stimulated with MDA-MB-231 derived EVs, were plated in 200 μL of IMDM supplemented with 20% EV-depleted FBS per well. Cells were maintained at 37 °C with 5% CO_2_. After 3 days in culture, 3 × 10^3^ CFSE-labeled human breast cancer cell lines were plated on the top of HSF spheroids. After 16, 24, and 48 h of coculture, the supernatants were harvested and spheroids were vigorously washed three times with PBS to remove non-adherent cells and cells attached to their surface, adding these cells to the previously harvested supernatant. Spheroids were mechanically (pipetting) and enzymatically dissociated with 0.25% trypsin (0.78 mM EDTA). Each experiment was performed in triplicate, wherein each replicate contained a pool of three or four spheroids and their respective supernatants, separately. Each sample was washed once and resuspended rigorously in 400 μL of PBS containing 3% FBS and 0.1% monosodium azide. The content of CFSE^+^ cells in the supernatant and spheroids was evaluated by flow cytometry, determining the acquisition parameter as the time of 150 s. The proportion of cells that invaded the spheroids was calculated as the percentage of the total. After 48 h, HSF spheroids cocultured with CFSE-labeled MCF-7 cells were vigorously washed as described above, fixed, and processed for confocal microscopy as previously described [63].

### 4.10. Immunofluorescence

HSF, stimulated or not, were distributed on coverslips in a 24-well plate with IMDM supplemented with 10% EV-depleted FBS at a concentration of 15 × 10^3^ cells per well and cultivated for 72 h. Then, they were washed twice with PBS e fixed with 2% paraformaldehyde for 30 min. Cells were washed twice again and permeabilized with 0.25% Triton X-100 (Merck, Rahway, NJ, USA) for 10 min. After that, they were washed three times and incubated in a solution containing 1% BSA and 0.25% Triton for 30 min. Then, they were washed once with PBS and incubated for 16 h at 4 °C with primary unlabeled antibodies anti-FAP and anti-αSMA diluted in PBS containing 1% BSA and 0.125% Triton, washed three times with PBS and incubated for 1 h at room temperature with conjugated secondary antibodies diluted in PBS with 1% BSA. Then, the coverslips were washed three times, incubated with DAPI for 10 min and washed again. Slides were mounted with N-propyl-gallate and images were examined and captured after instrument setup using a Leica TCS-SPE Confocal Microscope. For each condition, were taken images from three random fields at 400× magnification. Mean fluorescence intensity was evaluated by selecting at least five regions of interest (ROI) per field of three independent experiments, using the Leica Application Suite X Software v. 3.7.4 quantification tool. Cell nuclei per sample were quantified and fluorescence intensity was expressed as the mean per cell. The fluorescence intensity of α-SMA and FAP in regions of interest was adjusted to the percentage of control.

### 4.11. Nuclear Magnetic Resonance Analysis

Pure IMDM, medium with ASA, and the conditioned medium of MDA-MB-231 cells, treated or not with ASA, obtained at 24 h and 24 + 24 h conditions were collected and centrifuged at 1600× *g* for 20 min (Sorvall Legend RT centrifuge–Thermo Fisher) to remove cellular fragments. Then, the samples were lyophilized and resuspended in 600 µL of deuterated water. ^1^H NMR spectra were acquired on a Varian VNMRS 500 to 499.77 MHz spectrometer on a multinuclear probe of 5 mm at 25 °C, with 128 accumulations, and processed with the MeReNova 10.1 program.

### 4.12. RNA Extraction and Real-Time Quantitative Polymerase Chain Reaction (qRT-PCR)

Total RNA from stimulated or not HSF was obtained using the mirVana™ miRNA Isolation Kit (Thermo Fisher) according to the manufacturer’s recommendations. The obtained RNA was quantified by spectrophotometry (NanoDrop™ Lite, Thermo Fisher) and 200 ng total RNA of each sample condition was reversely transcribed to cDNA using High-Capacity cDNA Reverse Transcription Kit (Thermo Fisher). Briefly, 10 μL of RNA (at a concentration of 20 ng/μL) was added to a mixture containing 4.2 μL of RNAse-free water, 2 μL of enzyme buffer 10×, 2 μL of random primers 10×, 0.8 μL of nucleotides 25× and 1 μL of MultiScribeTM Reverse Transcriptase enzyme. This mix was incubated according to the following: 25 °C for 10 min, 37 °C for 120 min, 85 °C for 5 min, and reserved at 4 °C indefinitely.

The mRNA expression was assessed by qRT-PCR using Power SYBR™ Green PCR Master Mix 1× (Thermo Fisher, Waltham, MA, USA) performed in a 96-well StepOnePlus Real-Time PCR System (Applied Biosystems, Waltham, MA, USA)). Specifically, was used 5 ng of cDNA, 200–300 nM of each primer, and the SYBR™ Green Mix to the final reaction volume of 15 µL. Samples were incubated for 10 min at 95 °C and then cycled at 15 s annealing at 95 °C, and 1 min extension at 60 °C, repeated 45 times. The relative gene expression was measured using GAPDH as a housekeeping gene. The primers used were MMP2-F: 5′AGCTCCCGGAAAGAGTTGATG3′; MMP2-R: 5′CAGGGTGCTGGCTGAGTAGAT3′; MMP14-F: 5′GCAGAAGTTTTAAGGCTTGCA3′; MMP14-R: 5′TCGAAGATTGGCCTTGATCTC3; GAPDH-F: 5′TGCACCACCAACTGCTTAGC3′; GAPDH-R: 5′GGCATGGACTGTGGTCATGAG3′.

### 4.13. Statistical Analysis

Nonparametric and parametric ANOVA statistical tests with post-test or Student’s t-test were performed, according to data distribution. Statistical significance was considered when *p* < 0.05. When necessary, statistical information was presented below the data. The software GraphPad Prism^®^ version 6.01 was used as a tool for analysis.

## Supplementary Materials

The following supporting information can be downloaded at: https://www.mdpi.com/article/10.3390/ijms241512020/s1.
