# Supplementary material for: Aspirin Affects MDA-MB-231 Vesicle Production and Their Capacity to Induce Fibroblasts towards a Pro-Invasive State
Source: Int J Mol Sci. 2023 Jul 27;24(15):12020. doi: 10.3390/ijms241512020 (PMC10419278; doi:10.3390/ijms241512020)
Supplement: Supplementary file 1 [file ijms-24-12020-s001.zip › ijms-2507393-supplementary.pdf]

Supplementary Figures and legends

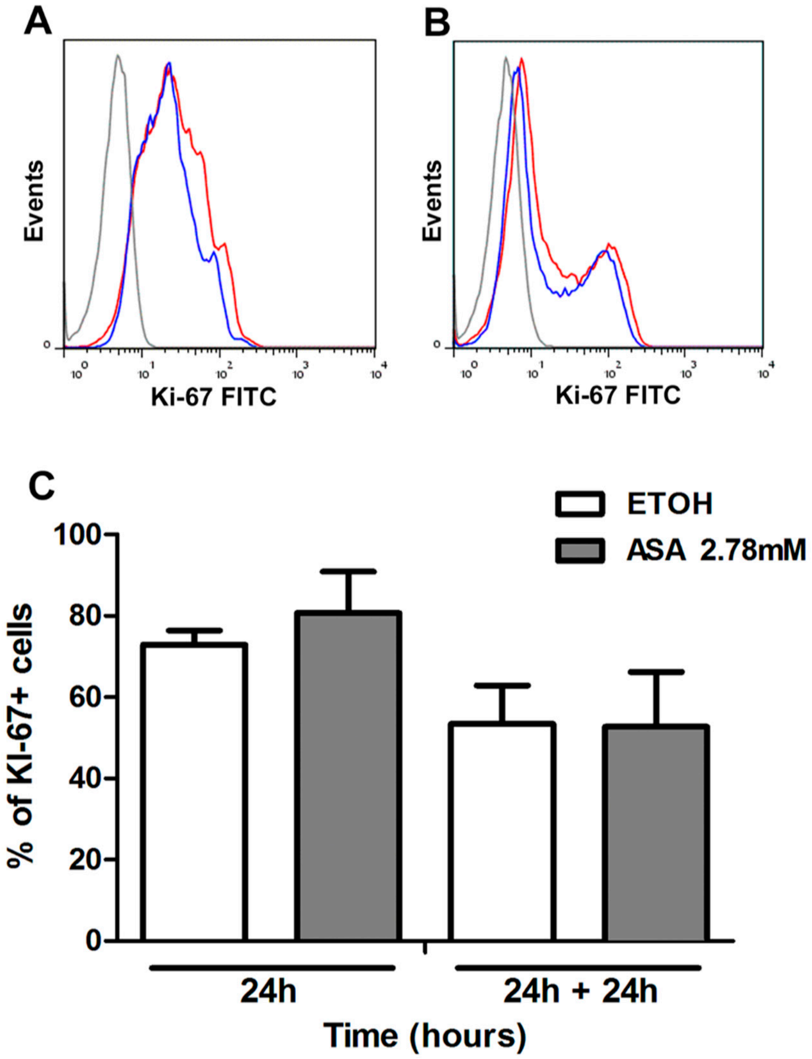

Supplementary Figure S1. Aspirin does not affect MDA-MB-231 proliferation. Breast cancer cell line MDA-MB-231 were treated for 24 hours (24 h) with 2.78mM of ASA or ETOH (vehicle) and then maintained for 24 h in the absence of ASA (24+24h). The percentage of cells expressing the cell cycle nuclear antigen Ki-67 was evaluated by FACS analysis. (A-B) Histograms showing Ki-67 fluorescence in MDA-231 after 24 (A) and 24+24 (B) hours. Red lines = ASA treated MDA-MB-231 cells, blue lines = ETOH control cells, gray lines = isotype control antibody. (C) Percentage of Ki-67^{+} cells. Data show mean ± SEM of 3 independent experiments

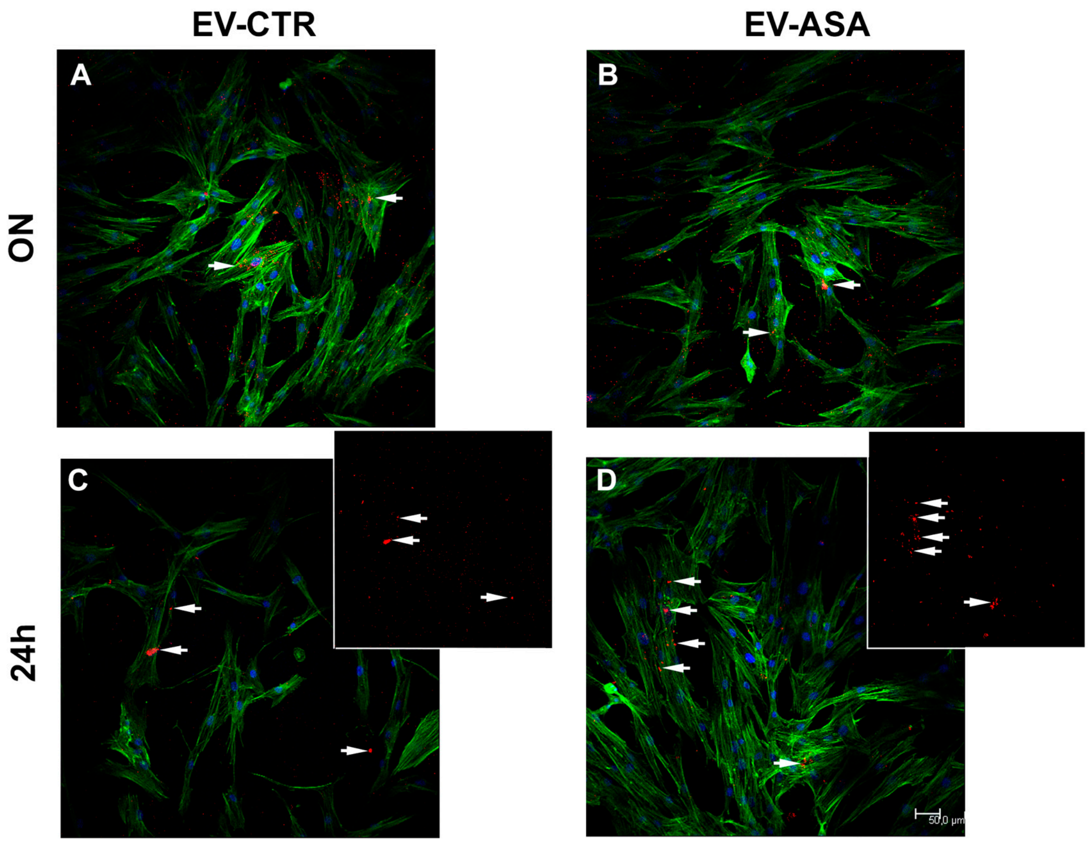

Supplementary Figure S2. Fibroblasts capture EVs derived from both ASA treated or not MDA-MB-231 tumor cells. Fibroblasts were cultured for up to 24 h with EVs derived from Vybrant DiI-labeled (red) MDA-MB-231 cells that were treated with 2.78mM of ASA (EV-ASA) or not (EV-CTR). (A-D) Fibroblasts cultured overnight (ON) (A, B) and 24h (C, D) with EV-CTR (A, C) and EV-ASA (B, D). The arrows show DiI+ punctiform pattern (red), indicating the presence of labeled-EV clusters in the HSF cytoplasm. Cytoskeleton was detected by phalloidin staining (green) and DAPI stained nuclei (blue). Inserts in C and D show DiI staining alone. Size bar = 50 μm.

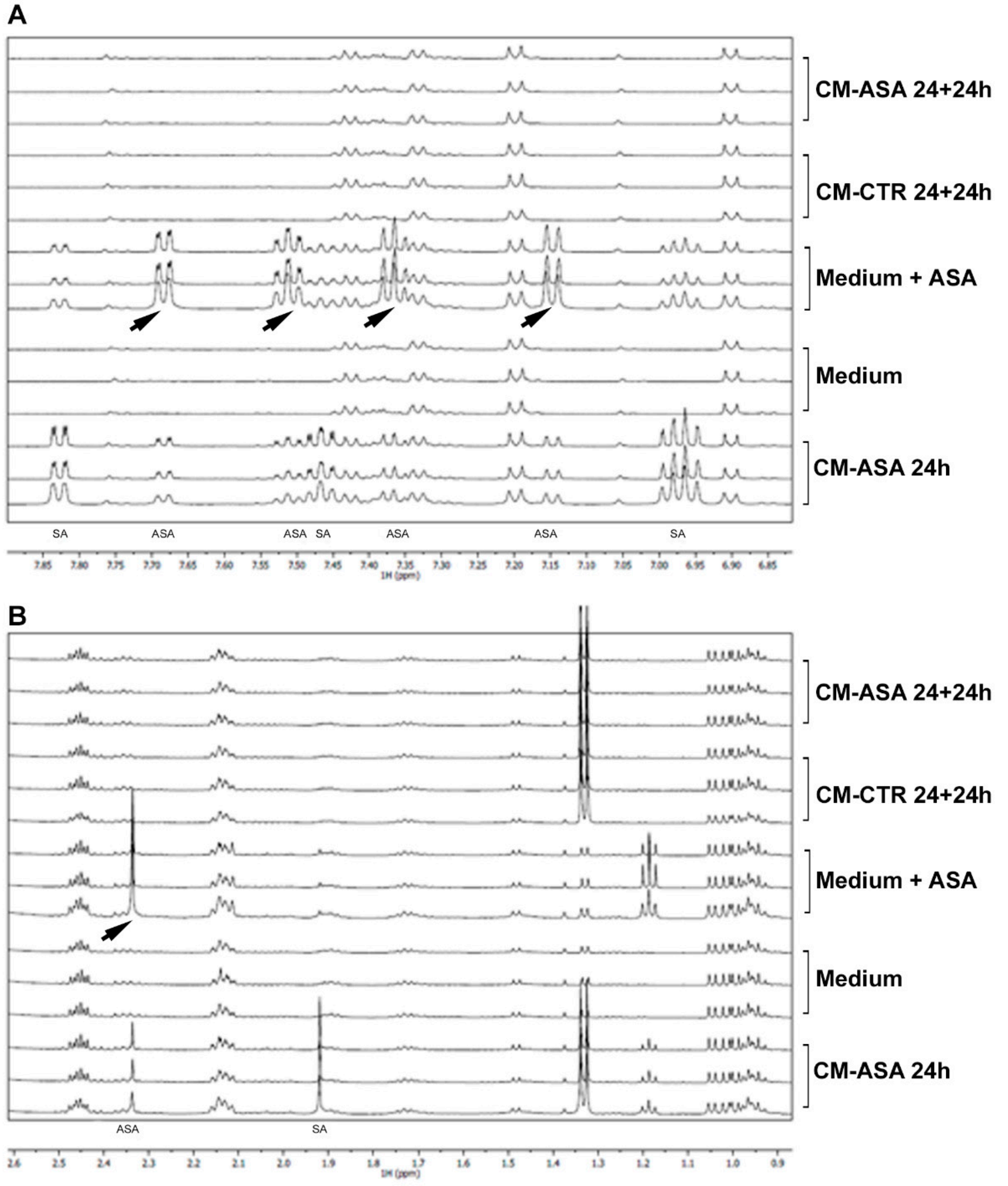

Supplementary Figure S3. ASA is hydrolyzed by MDA-MB-231 cells and no trace of ASA or SA is observed in the CM after 24 hours. Representative analysis of the conditioned medium of ASA-treated (CM-ASA) or not (CM-CTR) MDA-MB-231 cells collected and analyzed by ^{1}H NMR. Data show the aromatic (A) and aliphatic (B) hydrogen NMR profile of ASA and the product of its hydrolysis, salicylic acid (SA). Arrows indicate ASA peaks. ASA: aspirin; SA: salicylic acid; Medium: pure IMDM; Medium + ASA: IMDM with 2.78 mM of ASA; CM-ASA 24h: conditioned medium of MDA-MB-231 incubated with 500 μg/mL of ASA for

24 hours; CM-ASA 24+24h: conditioned medium of MDA-MB-231 incubated with 500 µg/mL of ASA for 24 hours and cultured for more 24 hours after ASA removal; CM-CT 24+24h: conditioned medium of MDA-MB-231 with no treatment at the time of 24+24h. Experiment was conducted in triplicate.

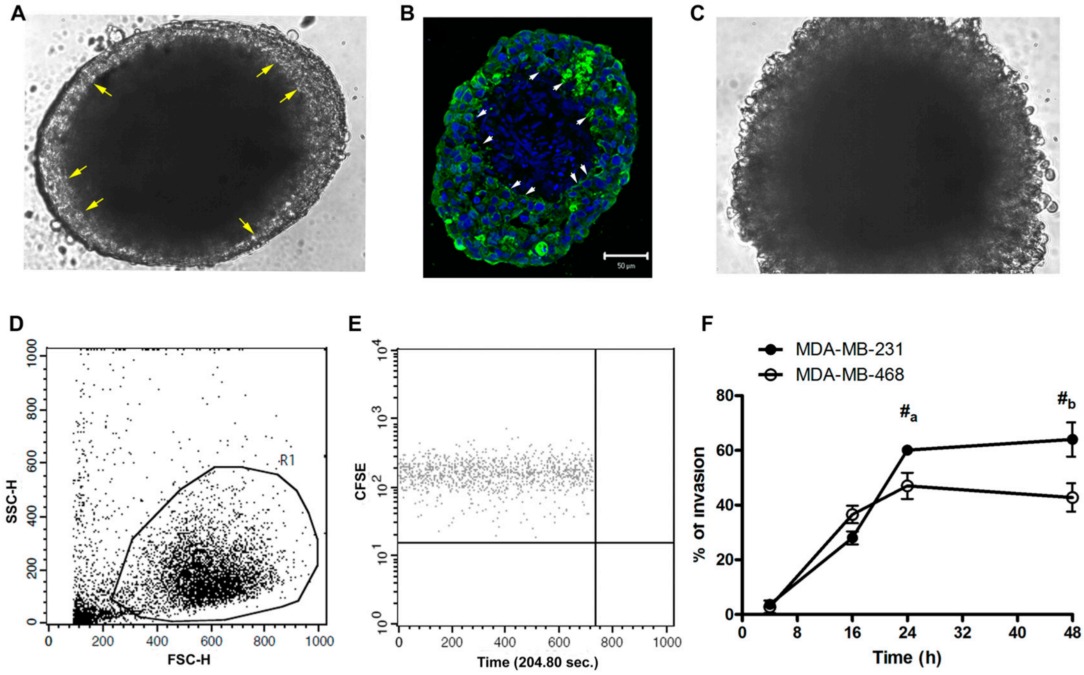

Supplementary Figure S4. Model of spheroid fibroblast invasion. (A-C) Breast cancer cell lines T-47D (A), MCF-7 (B), both representative of non invasive luminal cancer, and MDA-MB-231(C), representative of invasive triple negative cancer, were cocultured with fibroblast spheroids for up to 72h. (A, C) Contrast phase microscopy. Original magnification = 100X. (B) Confocal microscophy. MCF7 cells are CFSE-labeled. Nuclei are stained with DAPI. Note the nitid boundary between noninvasive tumor cells and fibroblasts (yellow and white arrows in A and B) indicating no invasion that is not observed in MDA-MB-231 coclutured with fibroblast spheroid (C). (D-F) Cytofluorimetric analysis of CFSE labeled MDA-MB-231 co-cultured for 24 h with unstimulated (control) spheroid

fibroblasts. (F) Invasion kinetics of MDA-MB-231 and MDA-MB-468 (both triple negative tumor cell lines cells) into the spheroids.
